# Effect of Different Types of “Dry Way” Additions in Porous Asphalt Mixtures

**DOI:** 10.3390/ma15041549

**Published:** 2022-02-18

**Authors:** Pedro Lastra-González, Jorge Rodriguez-Hernandez, Carlos Real-Gutiérrez, Daniel Castro-Fresno, Ángel Vega-Zamanillo

**Affiliations:** 1 GITECO Research Group, Universidad de Cantabria, Av. de los Castros 44, 39005 Santander, Spain; rodrighj@unican.es (J.R.-H.); carlos.real@unican.es (C.R.-G.); castrod@unican.es (D.C.-F.); 2 GCS Research Group, Universidad de Cantabria, Av. de Los Castros 44, 39005 Santander, Spain; angel.vega@unican.es

**Keywords:** SBS, rubber, ELT, polymer, asphalt, modified bitumen, dry method

## Abstract

Polymers are widely used to improve the mechanical performance of asphalt mixtures. Among them, styrene butadiene styrene (SBS) is the most commonly used, especially in the wet modification of virgin bitumen. This method, which is extensively utilized, has several advantages, but also some disadvantages, concerning its performance (such as the risk of instability or a lack of homogeneity) and logistical management (such as the need for special equipment, the transport of materials, and the dependence on the refinery that modifies the bitumen). This paper analyses the use of the most conventional types of polymers (two types of SBS, one type of BS, and rubber from end-of-life tires), dry added, as an alternative method. They have been used in porous asphalt mixtures. This type of bituminous mixture is usually designed with commercial polymer-modified bitumen, due to the mechanical requirements, and it is very sensitive to the properties of the binder used. The mechanical behavior of experimental porous asphalt mixtures has been significantly improved, especially in the case of SBS, although the performance did not reach that of commercial polymer-modified bitumen. The results have shown that the dry method is a suitable and feasible option to manufacture modified mixtures, especially considering its advantages, from a logistical viewpoint, in comparison with the wet method.

## 1. Introduction

Polymer-modified bitumen (PMB) is obtained by applying polymers to a virgin binder (VB) to improve several properties and reduce some of the traditional failure mechanisms of bituminous mixtures [1]. The implementation of PMB is widely established, and several providers offer this type of bitumen, which is principally modified with different types of polymers, including styrene butadiene styrene (SBS), acrylonitrile butadiene styrene (ABS), ethylene-vinyl acetate (EVA), or ethylene-butyl acrylate (EBA), as well as others, such as rubber from end-of-life tires (ELT) [2,3,4,5].

Among these, SBS is the most widely used polymer. This tri-block polymer is made up of rigid nodes of styrene, which are interconnected by flexible chains of butadiene [6]. The strength and elasticity of SBS are directly related with the physical cross-linking of the molecules, producing a three-dimensional network [7]. Recently, new modifiers have been developed, in order to improve the mechanical and environmental properties of PMB in particular, and bituminous mixtures in general, such as different types of SBS with different structures and modified with chemical compounds [8,9,10], plastic waste [2,11,12], or nanomaterials [13,14].

The most common addition technique is the wet method. In this case, the additives are directly added to the raw binder [15]. This process usually requires high temperatures, and different mixing energies are applied to achieve the reaction of these additives with the bitumen. Despite the clear improvements that this process achieves in the mechanical behavior of the binder, it has the following limitations: it requires the use of special equipment, the transport of materials to and from the refinery that modifies the bitumen, and the resulting modified bitumen can present problems, such as instability or a lack of homogeneity [6,16,17,18].

Another option, which is less extended, is the incorporation of the modifiers directly into the bituminous mixture in the mixer drum; this is known as the dry method [15]. In this case, the additives are not pre-mixed with the binder; they are directly added in the asphalt plant, which adapts the manufacturing process of the complete asphalt mixture to their incorporation. Then, there is a reaction between the additive and the binder, but this reaction is much weaker than in the wet process, and quality control should be performed directly on the final mixture. The additives are usually added by a doser and the mixing time should be slightly increased. This method, which has mainly been used with rubber as a modifier, is much less studied, even though it is easier to implement [16]. The dry method enables the modifiers to be stored until they are required, increasing the time period for which the modifier is available. Moreover, the impact of transportation is lower, and, in the case of rubber, the cost efficiency is improved [19]. However, this method also has some drawbacks, such as the irregular performance if the manufacturing and lay-down processes are not well controlled [19], due, fundamentally, to the volume instability because of the expansion of rubber [20]. The use of different modifiers by the dry method is minimal in comparison with rubber or plastic waste polymers, which have been mainly assessed by the dry method, in order to improve the environmental impact of roads [21,22,23,24]. In the case of SBS, it is the most common polymer used by the wet method to provide good elastic properties to raw bitumen [25]; however, its use by the dry method is very limited, due to the improvements it achieves by the wet method [26]. The latest advances have analyzed SBS as one of the materials that constitutes a modifier, together with other additives, such as waste rubber or reclaimed plastic. This new additive improves some properties, such as rutting resistance [27]. SBS has also been used as pellets by the dry method, under heavy traffic and severe weather conditions, in a project in Greenland, with the aim of increasing the flexibility and rutting resistance of traditional roads [28].

This paper reports the performance of different types of additions by the dry method. A porous asphalt mixture was selected to test the performance of various additions because this type of mixture is usually designed with a PMB, as it undergoes the highest mechanical stresses and strains, due to the high percentage of voids. Different types of BSs (including SBS polymers) were analyzed, incorporating them directly into the mixer drum at different temperatures. Furthermore, a mixture modified with ELT rubber was also included in the research, to provide better knowledge of the impact of different polymers, regarding the mechanical behavior of the mixtures. The results were compared with a commercial PMB and a conventional raw binder, both used in reference mixtures.

## 2. Materials and Methodology

### 2.1. Materials

The aggregates used were the conventional aggregates employed to manufacture roads in the north of Spain, which are ophite and limestone. Ophite (a type of porphyry igneous rock) was used in the coarse fraction, and limestone was used in the fine and filler fractions. The properties of both types of aggregates are detailed in Table 1.

Bitumen with the conventional penetration grade 50/70 was used to design the first reference and the experimental mixtures with the additions. Furthermore, the polymer-modified bitumen PMB 45/80-65 was used for the second reference mixture. Consequently, the first reference represents a baseline to quantify the influence of the polymers added by the dry method; meanwhile, the second reference represents the objective to be reached by the experimental mixtures. The properties of both binders are shown in Table 2 and Table 3.

Four polymers were used as modifiers of the conventional bitumen, including two different types of copolymer SBS, the same copolymer, but with two BS segments, and rubber from end-of-life tires (ELT). The different properties of each of them are reported in the Table 4.

### 2.2. Specimen Preparation

**Figure 1:**
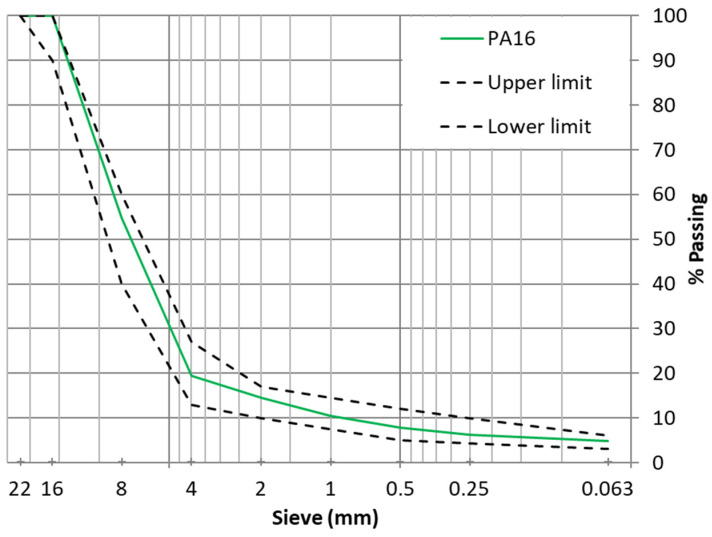
Particle size distribution of asphalt mixtures.

The same particle size distribution (Figure 1) and binder content (4.5% above mixture) were used for every porous asphalt mixture.

The asphalt samples were produced by attempting to replicate, as much as possible, the conventional manufacturing process. In the case of the reference mixtures, the following manufacturing temperatures were selected according to the recommendations of the providers of the bitumen: 150 °C for the conventional 50/70 penetration grade binder, and 170 °C for PMB 45/80-65. The mixture with conventional bitumen and rubber ELT was produced at 170 °C, since it is well known that this additive increases the viscosity and requires partial digestion with bitumen. The experimental mixtures with virgin rubber polymer (the two SBS and the BS) were manufactured at both temperatures (150 and 170 °C) to check the influence of temperature on their mechanical behavior. A polymer concentration of 6% was chosen, since this percentage is usually applied in the wet process [29,30]. Table 5 presents the experimental and reference mixtures considered in this study.

The additives were introduced just after the binder, directly into the mixer drum, and, therefore, they were mixed at the same time with the aggregates.

### 2.3. Testing Program

To compare the influence of the different additives on the mechanical characteristics, the experimental mixtures were designed by incorporating 6% of each of them by the total weight of bitumen. This percentage is in line with previous studies, in which the additives were added by the wet method [18]. The tests outlined in the following sections were performed to study the main properties of the PA mixtures.

#### 2.3.1. Voids Test (EN 12697-8)

The internal structure plays a crucial role in the mechanical and functional performance of PA mixtures. The total air voids were measured based on the volumetric determination test, according to European standard. Eight samples of each mixture were considered.

#### 2.3.2. Binder Draindown Test (EN 12697-18)

This test was performed by adding an uncompacted sample to a normalized mesh basket. The test was carried out 25 °C above the mixing temperature in the case of the conventional binder, and 15 °C above the manufacturing temperature for PMB. The binder drainage is expressed as the percentage of bitumen draindown from the mass of the uncompacted mixture after 3 h, according to the following equation:

(1) $$Binder\;drainage\;\left(\%\right)=\frac{b}{m}$$

where *b* (g) is the quantity of binder separated and *m* (g) is the mixture mass.

#### 2.3.3. Cantabro Particle Loss Test under Dry (EN 12697-17) and Wet Conditions (NLT 362/92)

The Cantabro particle loss test was carried out with 4 samples per condition. It assesses the raveling resistance of the porous asphalt mixtures by measuring the percentage of mass loss that takes place when the specimen is subjected to abrasion in the Los Angeles machine. The Cantabro test was also performed in wet conditions; in this case, the specimens were submerged in water at 60 °C for 24 h, as a method to evaluate the water damage. Then, the samples were kept at 25 °C for 24 h again before performing the test. In both cases, the loss in mass is expressed as the percentage after 300 turns and is calculated according to the following equation:

(2) $$Particle\;loss\;\left(\%\right)=\frac{m_{i}-m_{f}}{m_{i}}$$

where *m_i_* (g) is the initial mass and *m_f_* (g) is the final mass.

#### 2.3.4. Water Sensitivity Test (EN 12697-12)

This test was performed with 8 samples. The water damage was measured by dividing the samples into two groups. One group of specimens was stored at room temperature (20–25 °C), while the other was vacuumed (6.7 ± 0.3 kPa in 10 min) and then immersed in water at 40 °C for 72 h. Once conditioned, both groups were brought to the test temperature (15 °C), and the indirect tensile strength (ITS) was calculated.

The water damage is calculated with the following equation:

(3) $$I.T.S.R.\;\left(\%\right)=\frac{ITS_{w}}{ITS_{d}}$$

where *I.T.S.R.* is the indirect tensile strength ratio, *ITS_w_* (kPa) is the indirect tensile strength of conditioned samples in wet conditions, and, finally, *ITS_d_* (kPa) is the indirect tensile strength of the dry group.

The results were statistically analyzed with Minitab software to determine whether the differences in mechanical behavior were significant. In all cases, 95% confidence interval (*p*-value 0.05) was applied. The Student’s t-test was employed when normal distribution of the results and homogeneity of the variances were observed, and the Mann–Whitney U test was used otherwise.

## 3. Results

### 3.1. Voids and Binder Draindown Tests

Table 6 presents the results of the voids test, which represents the structure of the PA mixtures. Taking into consideration that every mixture has the same particle size distribution, bitumen quantity, and the same manufacturing temperature as one of the reference mixtures, the differences can be exclusively attributed to the impact of the type of additive.

Both reference mixtures show a significant difference in the percentage of voids, despite having been manufactured at the temperature recommended by the supplier. The probable reason for this is that the viscosity increases faster in the case of the PMB mixture, when it is cooled down after the manufacturing process, because this type of bitumen requires a higher temperature, increasing the percentage of voids [31,32]. The impact of the manufacturing temperature on the void content of the experimental mixtures is low; the results are quite similar when we compare both temperatures for the same type of polymer. However, what is remarkable is the impact of the SBS type D polymer and BS in relation with the 50/70 reference mixture. The statistical analysis (Table 7) shows that both additions significantly increase the percentage of voids, reaching the same percentage of voids as the objective reference mixture with PMB. On the other hand, the SBS A polymer and the ELT rubber offer the same percentage of voids as the conventional bitumen reference.

None of the mixtures presented binder draindown.

### 3.2. Cantabro Test under Dry and Wet Conditions

Raveling was measured by the Cantabro test. The test was performed in both dry and wet conditions; therefore, the damage due to water action was also considered when the cohesion of the mixtures was analyzed. Table 8 presents the results of the test.

The manufacturing temperature influences the cohesion of the mixtures, with the experimental mixtures that were manufactured at 170 °C showing better results. This is probably linked with the better digestion of the additives achieved by the bitumen at a higher temperature.

**Figure 2:**
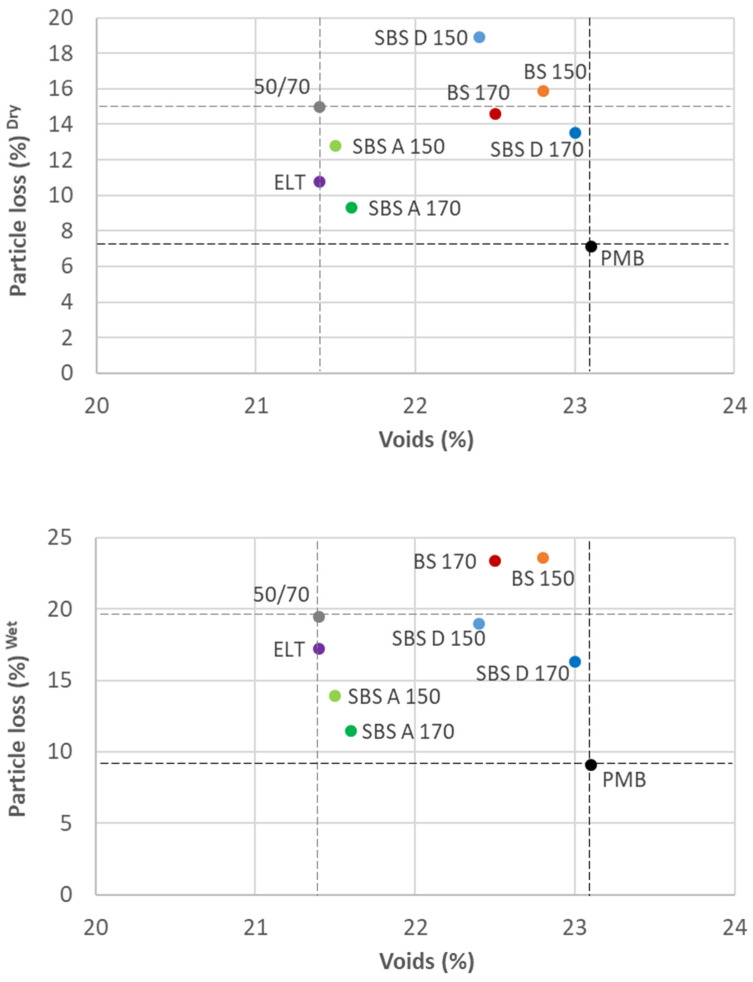
Cantabro test under dry (**top**) and wet (**bottom**) conditions.

The percentage of voids of each mixture should be considered to properly understand the impact of each polymer, because voids have a huge influence on particle loss. The lower the percentage of voids, the lower the particle loss normally is. Consequently, it must be considered that the SBS D polymers and BS significantly increased the percentage of voids up to the level of the mixture with PMB. Figure 2 shows the particle loss in relation to the voids under dry and wet conditions.

In general, additives decreased the particle loss in comparison with the 50/70 reference mixture, especially both types of SBS at 170 °C. SBS A, which, statistically, has the same percentage of voids as the 50/70 reference mixture, has significantly lower particle loss under dry and wet conditions (Table 9 shows the *p*-values for the experimental mixtures in relation to the references). Moreover, its results are not statistically different than those obtained with the PMB reference mixture (although the former has a lower percentage of voids). In the case of SBS D, the mixture slightly improves the result of the reference with the conventional binder at 170 °C, but offers a higher percentage of voids. The behavior of BS is similar under dry conditions, but it works worse under wet conditions. The rubber from ELT also improves the performance of the 50/70 reference mixture, but only significantly under dry conditions.

In general, all additives decrease the particle loss in comparison with the 50/70 reference mixture, especially under dry conditions. This improvement is significant with both types of SBS at 170 °C, independently of the conditions. Despite this, none of the polymers achieve the performance of the PMB.

### 3.3. Water Sensitivity Test

The water damage was analyzed by measuring the indirect tensile strength (ITS) of all the samples under dry and wet conditions. As with the Cantabro test, the percentage of voids has a great influence on these results, because voids facilitate the penetration of water into the samples. The ITS results are shown in Table 10. Better results were reached by increasing the manufacturing temperature, which is coherent with achieving better digestion of the polymers by the binder.

The addition of polymers by the dry method clearly increases the resistance of the samples, especially for both SBS polymers. Although the ratio of the experimental mixtures (I.T.S.R.) is decreased, this does not imply poorer behavior, because the significant increase in the resistance under dry conditions even exceeds the resistance of the reference mixture with PMB, with the same proportion of voids (as the SBS D at 170 °C).

**Figure 3:**
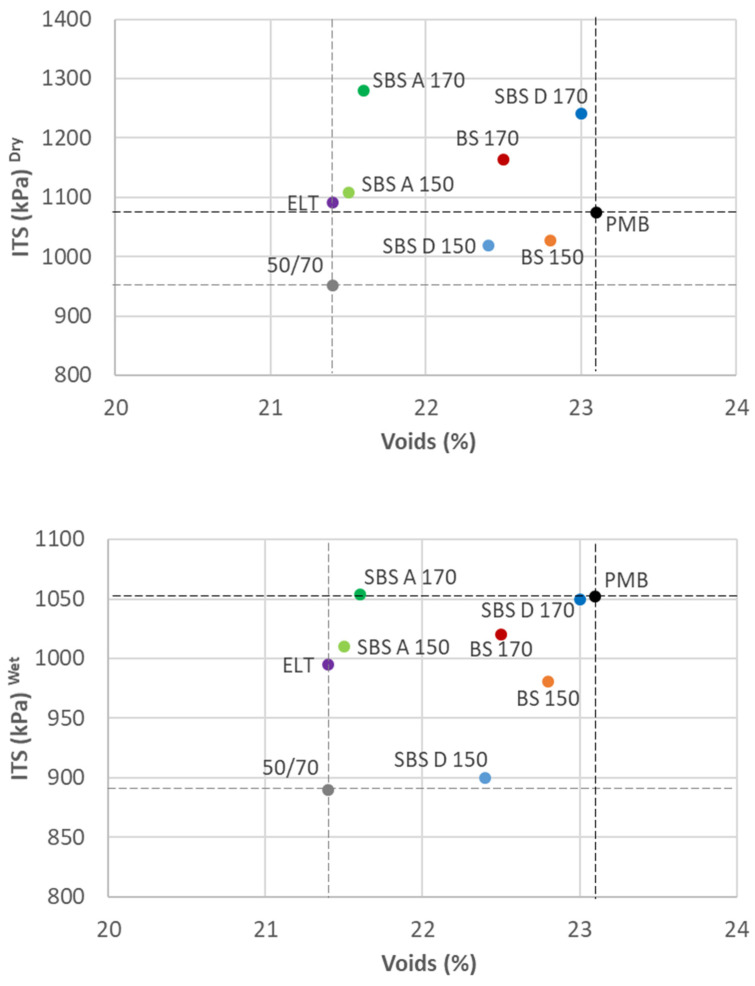
Indirect tensile strength under dry (**top**) and wet (**bottom**) conditions.

As in the case of the Cantabro test, the results plotted against the percentage of voids for each mixture are shown in Figure 3, under dry and wet conditions.

The *p*-values used to analyze the results are presented in Table 11. In this case, some of the polymers (such as SBS A at 170 °C in dry conditions) are statistically different to the reference mixture with PMB, exceeding its resistance. However, in most of the cases, there are no significant differences between the mixtures modified with the polymers by the dry method and the mixture with the commercial PMB. It is worth mentioning the behavior of the mixture with SBS D at 170 °C; it reaches higher resistance than the PMB reference mixture, with the same percentage of voids, under dry conditions, and this resistance is the same under wet conditions.

## 4. Conclusions

Different types of polymers were added, by the dry method, to porous asphalt mixtures to analyze their influence on the mechanical characteristics. Two binders, conventional bitumen with 50/70 penetration grade and PMB, were used as references to quantify the effect of the additions on the mixtures. They were added to the mixer drum at the following manufacturing temperatures: the same temperature as the conventional 50/70 penetration grade bitumen and the same temperature applied to PMB. The incorporation of these additives modified the structure of the PA reference mixture, increasing the percentage of voids, so the impact of the polymers was analyzed by considering this variable, because it has a great influence on the mechanical behavior of PA mixtures.

The following conclusions were drawn from the discussion of the results:

- The polymers can be added at the same temperature as the reference mixture with the raw binder, and the behavior will be improved. However, better performance is obtained when the temperature is increased up to similar values to those used for the manufacturing of PMB.
- The impact on the structure of the PA mixture depends on the type of polymer; while SBS A and ELT do not modify the void percentages, SBS D and BS increase them.
- All polymers added by the dry method improve the resistance to raveling of the 50/70 reference mixture. The best results were achieved by both SBSs, which significantly decreased the particle loss; however, their performances do not reach the behavior of the PMB.
- The indirect tensile strength is greatly increased by the addition of the polymers, achieving the same behavior as PMB, and significantly improving the performance of raw bitumen.

Consequently, the addition of the polymers by the dry method is a suitable option to manufacture modified mixtures, especially considering the advantages from a logistical viewpoint (storage, cost, transport, etc.). A life cycle assessment and life cycle cost analysis should be undertaken to study this option more profoundly.

## Figures and Tables

**Table 1:** Properties of aggregates.

Properties	Result	Limits	Standard
Ophite			
Los Angeles coefficient	13	≤20	EN 1097-2
Specific weight (g/cm^3^)	2.794	-	EN 1097-6
Polished stone value (PSV)	>56	≥50	EN 1097-8
Flakiness Index (%)	8	≤20	EN 933-3
Limestone			
Los Angeles coefficient	28	-	EN 1097-2
Specific weight (g/cm^3^)	2.724	-	EN 1097-6
Sand equivalent	78	>55	EN 933-8

**Table 2:** Properties of 50/70 bitumen.

Test	Result	Standard
Penetration (25 °C, dmm)	57.0	EN 1426
Softening point (°C)	51.5	EN 1427
Density (g/cm^3^)	1.035	EN 15326
Fraass Point (°C)	−11	EN 12593

**Table 3:** Properties of PMB 45/80-65 bitumen.

Test	Result	Standard
Penetration (25 °C, dmm)	49.5	EN 1426
Softening point (°C)	72.5	EN 1427
Density (g/cm^3^)	1.028	EN 15326
Fraass Point (°C)	−13	EN 12593
Elastic recovery (25 °C, %)	90.0	EN 13398

**Table 4:** Properties of polymers used as additives.

Polymer	SBS A	SBS D	BS	Rubber ELT
Size max. (mm)	6.3	6.3	8.0	0.6
Specific weight (g/cm^3^)	0.93	0.93	0.899	1.15
Type	Block copolymer	Block copolymer	Block copolymer	-
Provider	Dynasol	Dynasol	Asphalt Plant	Renecal

**Table 5:** Mixtures considered in the study.

Mixtures	Bitumen	Polymer Type	Polymer/Binder (%)	Manufacturing Temperature (°C)	Label
Reference 1	50/70	-	-	150	50/70
Reference 2	PMB	*	*	170	PMB
Experimental 1	50/70	SBS A	6.0	150	SBS A 150
Experimental 2	50/70	SBS A	6.0	170	SBS A 170
Experimental 3	50/70	SBS D	6.0	150	SBS D 150
Experimental 4	50/70	SBS D	6.0	170	SBS D 170
Experimental 5	50/70	BS	6.0	150	BS 150
Experimental 6	50/70	BS	6.0	170	BS 170
Experimental 7	50/70	ELT	6.0	170	ELT

* Information not provided by the supplier.

**Table 6:** Voids test.

	50/70	PMB	SBS A		SBS D		BS		ELT
Manufacturing T^e^ (°C)	150	170	150	170	150	170	150	170	170
Density (g/cm^3^)	2.032	1.992	2.035	2.031	2.011	1.997	2.001	2.009	2.036
Voids (%)	21.4	23.1	21.5	21.6	22.4	23.0	22.8	22.5	21.4

**Table 7:** *p*-values of voids test.

	SBS A		SBS D		BS		ELT
Manufacturing T^e^ (°C)	150	170	150	170	150	170	170
50/70	0.968	0.815	0.017	0.000	0.000	0.010	0.921
PMB	0.000	0.011	0.051	0.690	0.243	0.059	0.001

**Table 8:** Results of Cantabro test.

	50/70	PMB	SBS A		SBS D		BS		ELT
Manufacturing T^e^ (°C)	150	170	150	170	150	170	150	170	170
Particle loss ^Dry^ (%)	15.0	7.1	12.8	9.3	18.9	13.5	15.9	14.6	10.8
Particle loss ^Wet^ (%)	19.5	9.1	13.9	11.5	19	16.3	23.6	23.4	17.2

**Table 9:** *p*-values of Cantabro test.

		SBS A		SBS D		BS		ELT
Manufacturing T^e^ (°C)		150	170	150	170	150	170	170
50/70	Dry	0.345	0.010	0.125	0.508	0.618	0.845	0.015
	Wet	0.030	0.030	0.312	0.885	0.194	0.377	0.665
PMB	Dry	0.054	0.118	0.020	0.075	0.010	0.034	0.008
	Wet	0.030	0.665	0.030	0.030	0.030	0.051	0.030

**Table 10:** Results of water sensitivity test.

	50/70	PMB	SBS A		SBS D		BS		ELT
Manufacturing T^e^ (°C)	150	170	150	170	150	170	150	170	170
I.T.S. ^Dry^ (kPa)	951.9	1074.2	1108.5	1280.2	1019.5	1240.6	1027.4	1163.8	1091.0
I.T.S. ^Wet^ (kPa)	889.4	1052.2	1009.9	1053.6	899.7	1049.8	980.5	1020.5	994.7
I.T.S.R. (%)	93	98	91	82	88	85	95	88	91

**Table 11:** *p*-values of indirect tensile strength test.

		SBS A		SBS D		BS		ELT
Manufacturing T^e^ (°C)		150	170	150	170	150	170	170
50/70	Dry	0.194	0.030	0.665	0.061	0.195	0.030	0.112
	Wet	0.035	0.034	0.812	0.013	0.093	0.023	0.076
PMB	Dry	0.085	0.030	0.885	0.312	0.061	0.112	0.596
	Wet	0.385	0.891	0.017	0.958	0.184	0.496	0.286

## Data Availability

The data presented in this study are available on request from the corresponding author.
